# Implementation and evaluation of different eradication strategies for *Brachyspira hyodysenteriae*

**DOI:** 10.1186/s40813-020-00162-2

**Published:** 2020-09-14

**Authors:** Willem Neirynck, Filip Boyen, Ilias Chantziaras, Tamara Vandersmissen, Philip Vyt, Freddy Haesebrouck, Jeroen Dewulf, Dominiek Maes

**Affiliations:** 1 Department of Reproduction, Obstetrics and Herd Health, Faculty of Veterinary Medicine, Ghent University, Merelbeke, Belgium; 2 Present Address: Poulpharm, Prins Albertlaan, Izegem, Belgium; 3 Department of Pathology, Bacteriology and Avian diseases, Ghent University, Merelbeke, Belgium; 4 Animal Health Care Flanders, Hagenbroeksesteenweg, Lier, Belgium; 5 Dialab, Moortelhoekstraat, Belsele, Sint-Niklaas, Belgium

**Keywords:** Pigs, *Brachyspira hyodysenteriae*, Swine dysentery, Eradication

## Abstract

**Background:**

*Brachyspira* infections are causing major losses to the pig industry and lead to high antimicrobial use. Treatment of *Brachyspira (B.) hyodysenteriae* infections may be problematic due to the high level of antimicrobial resistance. The present study implemented and evaluated farm-specific eradication programmes for *B. hyodysenteriae* in 10 different infected pig farms in Belgium.

**Results:**

Ten pig farms clinically infected with *B. hyodysenteriae* volunteered to implement a farm-specific eradication programme. The programme depended on the farm and management characteristics, antimicrobial susceptibility of the *B. hyodysenteriae* strain and the motivation of the farmer. Two farms practiced total depopulation, six farms partial depopulation and two farms antimicrobial medication without depopulation. In addition, all farms implemented biosecurity measures, and faeces samples were tested for the presence of *B. hyodysenteriae* at 6, 9 and 12 months after the start of the program. Single *Brachyspira* isolates from before and after the programme were typed using multilocus sequence typing (MLST).

Eradication was successful in four farms. Two of them (farrow-to-finish and finishing herd) had applied total depopulation and respected a vacancy period of at least 3 weeks. A third farm (gilt farm) practised partial depopulation, the rooms remained empty for 28 days and changed the source of breeding gilts. The fourth farm practised partial depopulation, the stables remained empty for 3 weeks, and used antimicrobial medication. The eradication programme was not successful in six farms. Two of the latter farms only used medication without partial depopulation. Four farms practiced partial depopulation, one of them combined it with antimicrobial medication. The cleaning and disinfection procedures, rodent control, stand-empty period and/or other biosecurity measures in the six farms were not always implemented properly. In two of three farms, isolates belonging to the same MLST type were found before and after eradication.

**Conclusions:**

Total depopulation or partial depopulation combined with implementing strict biosecurity measures allowed eradication of *B. hyodysenteriae* from clinically infected pig farms. Programmes based on antimicrobials without depopulation or partial depopulation without strictly adhering to all suggested biosecurity measures were not successful. Stockmanship and motivation of the farmer to permanently maintain high biosecurity standards are essential for success.

## Background

Swine dysentery, caused by *Brachyspira hyodysenteriae (B. hyodysenteriae)*, is responsible for major health, welfare and production problems in the pig sector worldwide. Infections with *B. hyodysenteriae* may result in severe mucohaemorrhagic diarrhoea, decreased performance and elevated mortality rates [1]. Infected farms also incur losses for the implementation of treatment and control measures, and may face restrictions on selling animals to other farms.

Different antimicrobials can be used for treatment such as pleuromutilins (tiamulin and valnemulin), macrolides (tylosin and tylvalosin), lincosamides (lincomycin) and tetracyclines. As infected farms mostly remain infected for long periods of time, antimicrobial usage significantly increases on these farms. Apart from the costs, the high usage increases the selection for acquired antimicrobial resistance, not only in *Brachyspira* but also in commensals and (facultative) pathogenic bacteria of the pig. Acquired antimicrobial resistance in *B. hyodysenteriae* strains has been shown to be a problem for proper treatment of the disease. For this reason, the European Medicine Agency (EMA) does not recommend to use tylosin for the treatment of *B. hyodysenteriae* infections (EMA/492247/2014, Annex 2). During the last decade, decreased susceptibility has also been reported for tiamulin and valnemulin in several swine producing countries worldwide [2–7]. A new pleuromutilin resistance gene (*tva(A)* tiamulin valnemulin antibiotic resistance) has been identified which not only confers reduced pleuromutilin susceptibility but may also facilitate the development of higher-level resistance via mutations in genes encoding ribosome-associated functions [8].

No commercial vaccines are currently available. Different experimental inactivated [9–11] and attenuated [12, 13] vaccines have been tested but so far protective immunity has been limited. Three farms in this study used an autogenous vaccine. Literature data about the use of autogenous vaccines is very scarce and variable results have been reported. Neirynck et al. [14] could not show efficacy of an autogenous *B. hyodysenteriae* vaccine in a clinically diseased herd. Also, inactivated whole cell bacterins mostly induce protection against infection with a homologous serotype of *B. hyodysenteriae* [1].

Control measures in infected farms relate to improvements in management and biosecurity, efficient pest control, diet changes and the use of antimicrobial agents [15–17]. These measures may decrease the infection levels and reduce or prevent clinical signs, but they are generally not sufficient to eliminate the pathogen from the herd. This means that farms remain infected and incur performance losses. Therefore, it may be recommended to eliminate the pathogen from infected farms. Different strategies for elimination of *B. hyodysenteriae* have been reported [18–20]. These are based on total or partial depopulation of pig farms and/or antimicrobial medication of the entire herd, combined with improved management and biosecurity measures. Often eradication has been done only in one or a few herds, and the success rate has been variable [21, 22].

The present study aimed to implement and evaluate farm-specific eradication programmes in 10 Belgian pig farms infected with *B. hyodysenteriae*. Farms were monitored at 6, 9 and 12 months after the start of the programme, and factors associated with success or failure were described.

## Methods

### Selection of the farms

Belgian pig veterinary practices were informed by phone, email and during a scientific meeting at the veterinary faculty about the project and asked whether they had client farms infected with *B. hyodysenteriae* that would be willing to participate. In total, 50 infected farms were contacted by the veterinarians. Most of the contacted farms were not willing to participate because of financial considerations, the fact that clinical signs were mild or absent and the uncertainty about a successful outcome. When farms showed interest in the project and considered participating, the farmer and veterinarian were asked to provide all relevant historic health and production data of the farm for the last 12 months, as well as diagnostic laboratory information, especially in relation to *Brachyspira*. After that, a farm visit was made by the principal investigator and the herd veterinarian to explain the study and to discuss different eradication programmes that could be successful for the farm. After the meeting, the farmers had to make a final decision whether they agreed to participate. In the end, 10 farms were enrolled in the study between April 2017 and May 2018.

### Farm description and diagnostic information about *B. hyodysenteriae*

A general description of the farms and diagnostic information about *B. hyodysenteriae* before the implementation of the eradication programme are shown in Table 1. The farms were representative of other farms in Belgium in terms of size, management practices, biosecurity measures, housing conditions and nutrition [23]. Severe diarrhoea was present on all farms, except for farm 1 where the diarrhoea was mild and only a few animals were affected. In farm 3, the slaughterhouse had also reported problems with colitis lesions in the slaughter pigs.

**Table 1:** General description of the farms (*n* = 10) and diagnostic information about *Brachyspira hyodysenteriae*

	Farm number									
	1	2	3	4	5	6	7	8	9	10
Type of farm^a^	gilt rearing	farrow-to-finish	sow	fattening	farrow-to-finish	fattening	sow	sow	fattening	fattening
Farm size^b^	1050	160	480	430	125	9000	280	550	2600	2300
Purchase of animals	at 10 weeks	breeding gilts at 7 months	no	at 10 weeks	no	at 10 weeks	no	no	at 10 weeks	at 10 weeks
All-in/all-out at … level										
Site	no	no	no	yes	no	no	no	no	no	no
Barn	no	no	no	yes	no	yes	no	no	no	yes
Room	yes	yes	yes	yes	yes	yes	yes	yes	yes	yes
Type of feed										
Sows	meal	pellets	meal		pellets		meal	meal		
Fattening pigs / gilts	meal	meal		meal	meal	meal			meal	meal
*Brachyspira hyodysenteriae*^c^										
Detected by …	PCR	PCR, culture	PCR, culture	culture	PCR	culture	PCR, culture	culture	culture	culture
Other *Brachyspira* spp. (culture)^c^	*pilosicoli, murdochii, innocens*				*murdochii*	*murdochii*				

^a^ Gilt rearing: from 10 weeks until pregnancy testing; sow farm: pigs raised until 10 weeks of age

^b^ Farm size: number of gilts (gilt rearing), sows (sow farm / farrow-to-finish), fattening pigs (fattening)

^c^ Based on diagnostics during the last year prior to the study

The *B. hyodysenteriae* isolates that were obtained from the participating farms were tested for their susceptibility against three antimicrobials commonly used for the treatment of swine dysentery [1] namely tiamulin, valnemulin and tylvalosin (Table 4). The agar dilution test was used as described by Vyt and Hommez [26]. Isolates were considered resistant if MIC-values (μg/ml) were higher than 2, 2 and 32 for tiamulin, valnemulin and tylvalosin, respectively [24, 25].

### Description of the eradication strategies

The type of eradication strategy depended on the type of farm, the farm structure and management practices, the antimicrobial susceptibility of the isolated *B. hyodysenteriae* strain, and the preference of the pig farmer. An overview of the different eradication strategies and the additional biosecurity measures taken in the 10 farms are shown in Table 2.

**Table 2:** Measures taken in the 10 farms that participated in the *Brachyspira hyodysenteriae* eradication programme

	Farm number									
	1	2	3	4	5	6	7	8	9	10
**Type of farm**^**a**^	gilt rearing	FTF	sow	fattening	FTF	fattening	sow	sow	fattening	fattening
**Eradication programme**										
Start during the year^b^	M-J	M-J	J-A	M-J	J-A	A-M	J-A	S-O	S-O	S-O
Depopulation of animals	partial	total	none	total	None	partial	partial	partial	partial	partial
Min. vacancy (d) of rooms during eradication	28	21 (nursery); 42 (other)	3 (nursery); 10 (sows)	56	3 (fattening)	14	10	21	14	21
Antimicrobial medication	no	no	yes	no	yes (except old fattening pigs)	no	yes	yes	no	no
Duration of medication (days)	NA^c^	NA	30	NA	30	NA	30	42	NA	NA
Antimicrobial^d^			Tiamulin		Tiamulin		Tiamu-lin^e^	Tiamulin 28d, lincomycin 14d		
**External biosecurity**										
Purchase of animals (age in weeks)	yes (10)	yes (30)	no	yes (10)	No	yes (10)	no	no	yes (10)	yes (10)
Temporary stop of animal purchase	yes	yes	NA	yes	NA	yes	NA	NA	yes	yes
Change of origin farm after programme	yes	no	NA	no	NA	no	NA	NA	no	no
**Internal biosecurity**										
Emptying and cleaning manure pits	yes	yes	yes	yes	no	yes	yes	yes	yes	yes
Strict / successful rodent control	yes	yes	no	yes	no	yes	yes	yes	yes	no
Proper cleaning and disinfection	yes	yes	yes	yes	no	yes	yes	yes	yes	yes
Disinfection baths at entry of each barn	yes	yes	yes	yes	yes	yes	yes	yes	yes	yes
Separate equipment for each age group	yes	yes	yes	yes	no	yes	yes	yes	no	no
Separate boots for each age group	yes	yes	no	yes	no	yes	yes	yes	no	no
**Eradication successful**	yes	yes	no	yes	no	no	no	yes	no	no

^a^ Gilt rearing: from 10 weeks until pregnancy testing; FTF farrow-to-finish; sow farm: pigs raised until 10 weeks of age

^b^ Months: A-M April–May; M-J May–June; J-A July–August; S-O September–October

^c^ *NA* Not applicable

^d^ Via feed medication: tiamulin 8–10 mg/ kg BW; lincomycin 15 mg/kg BW

^e^ For 6 months after the programme, breeding gilts prior to insemination were treated with tylvalosin (5 mg/kg BW) via the feed

Farms 2 and 4 implemented total depopulation, farms 7 and 8 used partial depopulated with antimicrobial medication, farms 1, 6, 9 and 10 tried partial depopulation without antimicrobial medication, and farms 3 and 5 just used medication. Total depopulation implied that all animals were removed from the entire farm (site) for a specific period of time. Partial depopulation was used when animals remained on the farm (site), so the entire farm did not have a vacancy period during one specific period of time. It was however possible that some rooms or barns became empty while other rooms or barns were still occupied by animals. In case a *B. hyodysenteriae* strain was isolated showing acquired resistance to both tiamulin, valnemulin and tylvalosin (farms 2, 9 and 10), total depopulation was proposed as the strategy of first choice. However, only the farmer of herd 2 agreed to implement total depopulation. For herds 9 and 10, the farmers indicated that a total depopulation was not an option, mainly because of financial concerns. Partial depopulation of a herd was accomplished by temporary stopping the purchasing of animals (farms 1, 6, 9 and 10), practising a stricter culling policy of the sows (farms 1, 7 and 8), selling the piglets at weaning (farms 7 and 8), selling breeding gilts at a younger age (farm 1), or sending fattening pigs sooner to slaughter (farms 6, 9 and 10). The type of antimicrobials used for medication, the dosage and treatment duration were determined in agreement with the herd veterinarian, and are shown in Table 2.

External biosecurity measures taken during the eradication programme related to purchase of animals and access of visitors to the pig stables. In all farms that purchased animals, a temporary stop in purchase was practiced. One farm (farm 1) also changed the origin farm where the pigs were purchased. Strict hygiene measures for persons entering the stables (protective clothes and shoes on the farm, hand hygiene) were proposed for all farms.

In terms of internal biosecurity, the recommendations related to emptying and cleaning the manure pits, strict rodent control, inserting a vacancy period for rooms, proper cleaning and disinfection of stables, using disinfection baths at the entrance of each barn, and separate equipment and boots for the different age groups.

Additional measures such as nutritional changes and the use of an autogenous vaccine against *B. hyodysenteriae* were voluntary implemented by some farmers, but were as such not part of the eradication programme. Nutritional changes pertained to the addition of adsorbents (Vitadys®, Nuscience) (farms 1, 2, 5, 6 and 8) or organic acids (farms 3, 5, 7, 9 and 10) to the feed of all animals on the farm, or to specific age groups. An autogenous vaccine against *B. hyodysenteriae* was used in three farms, namely at onset of the fattening period (farms 6 and 9) and in breeding animals (farm 7).

### Sample size calculations

The sample size required to achieve a 90% level of confidence of population freedom was calculated separately for sows and for fattening pigs using hypergeometric approximation method [27]. For this, the test’s sensitivity and specificity, within-herd prevalence, prior confidence of population freedom, probability of introduction and an indicative population size were specified. The within-herd prevalence was set at 5% [28, 29]. The test’s sensitivity was set at 90% and the specificity at 100%. A prior 75% confidence of freedom was set for sows and a 5% probability of novel introduction of *B. hyodysenteriae*. Regarding fattening pigs, a prior 65% confidence of freedom was set for fattening pigs and a 1% probability of novel introduction of *B. hyodysenteriae*. The results of these calculations showed that sample sizes of 27 for sows and 36 for fattening pigs, respectively, were required to achieve a 90% confidence of freedom.

### Sampling protocol to monitor the infection status post-eradication

Once all measures for the eradication programme had been put in place, the farms were monitored for *B. hyodysenteriae* in the faeces and for the presence of clinical signs. Individual faecal samples were taken by the principal investigator or farmer after rectal stimulation at 6, 9 and 12 months after the start of the eradication programme. In addition, periodic visits monitoring clinical signs on the farms were made by the herd veterinarian and principal investigator for at least 2 years.

In the sow and farrow-to-finish farms, for each sampling moment, random samples were taken from nine sows (*n* = 9). To increase the likelihood of finding positive samples [30], the sows were mainly sampled at stressful moments such as around farrowing, shortly (1–4 days) after weaning, or after moving them to the group housing unit when they had been tested pregnant. In the fattening and farrow-to-finish farms, 12 fattening pigs were sampled for each sampling moment. In case fattening pigs of different ages were present on the farm e.g. beginning, halfway, end of fattening period, samples were taken from pigs of different ages.

In three farms (1, 8, 10), the farmers preferred to perform the sampling themselves at the end of the study, i.e. 1 year after implementation of the eradication programme. On farm 9, the sampling was also done by the farmer himself in order to avoid too many persons entering the stables and the risk of reinfection by the researcher.

The faecal samples were transported cooled in closed plastic recipients to the laboratory within 2 h after collection.

### Analyses of the faecal samples

The individual faecal samples were pooled for analysis into pools of 3 individual samples [31]. They were investigated using real-time PCR for the presence of *B. hyodysenteriae* (BactoReal® Kit *Brachyspira hyodysenteriae*, Ingenetix, Vienna Austria) and if asked by the farmer also for *Brachyspira pilosicoli* (*B. pilosicoli;* BactoReal® Kit *Brachyspira hyodysenteriae*, Ingenetix, Vienna Austria). The PCR tests detect the NADH oxidase gene (*nox*) gene of *B. hyodysenteriae* and *B. pilosicoli*, respectively [32].

The samples were also used for culture of *B. hyodysenteriae*. They were cultured within 16 h after sampling on selective plates consisting of Trypticase Soy Agar (TSA) (Sigma-Aldrich, St. Louis, MO, USA) supplemented with 5% sheep blood (E&O Laboratories, Bonnybridge, UK), 1% yeast extract (Becton Dickinson, Franklin Lakes, NJ, USA), 6.25 μg/ml vancomycin, 2400 μg/ml spectinomycin and 6.25 μg/ml colistin (all antimicrobial compounds from Sigma-Aldrich, St. Louis, MO, USA). Plates were anaerobically incubated for 3 days at 41.5 °C and then incubated at 37 °C till 10 days. Suspected colonies were identified by MALDI-TOF MS (Bruker, Biotyper). Isolates were purified by three to five subcultures on Trypticase Soy Agar (TSA) plates supplemented with 5% sheep blood and 1% yeast extract [33] and stored at − 70 °C in 300 μl of a medium consisting of 75 ml horse serum (Thermo Fisher Scientific, Carlsbad CA, USA) and 25 ml Brain Heart Infusion (BHI) broth (Bio-Rad, Hercules CA, USA) supplemented with 10% (w/v) glucose (Merck, Darmstadt, Germany) until further use. All isolates were phenotypically characterized by determination of beta-haemolysis, indole production, hippurate hydrolysis and the presence of α-galactosidase, α-glucosidase and β-glucosidase as described previously [34].

The *B. hyodysenteriae* isolates that were obtained were subsequently typed using multilocus sequence typing (MLST), and the antimicrobial susceptibility against tiamulin, valnemulin and tylvalosin was tested using the agar dilution test described above. MLST typing was only done in farms from which *B. hyodysenteriae* isolates could be obtained before and after the eradication program, allowing comparison of MLST types. The MLST scheme as described by Rasbäck et al. [35] was performed with modifications [36]. For all strains, sequences for genes encoding alcohol dehydrogenase (*adh*), alkaline phosphatase (*alp*), esterase (*est*), glutamate dehydrogenase (*gdh*), glucose kinase (*glpK*), phosphoglucomutase (*pgm*) and thiolase (*thi*) were determined and matched with the online MLST database (http://pubmlst.org/brachyspira/).

## Results

The results of the testing for *B. hyodysenteriae* after the implementation of the eradication programme are shown in Table 3.

**Table 3:** Results of pooled faecal samples (1 pooled sample = 3 individual faecal samples) testing for *B. hyodysenteriae* and other *Brachyspira* spp. after implementation of the eradication programme (*n* = 10 farms). Farms with successful eradication are marked in bold

	Farm number									
	1	2	3	4	5	6	7	8	9	10
Type of farm^a^	**gilt rearing**	**farrow-to-finish**	sow	**fattening**	farrow-to-finish	fattening	sow	**sow**	fattening	fattening
Depopulation	**partial**	**total**	none	**total**	none	partial	partial	**partial**	partial	partial
Medication	**no**	**no**	yes	**no**	yes (only sows)	no	yes	**yes**	no	no
*B. hyodysenteriae*										
Month 6	**0/3**	**0/3 (sows)** **0/4 (FP)**^**c**^	0/3 (sows) 3/3 (FP)	**0/4**	0/3 (sows) 1/4 (FP)	3/4	0/3	**0/3**		
Month 9		**0/3** **0/4**		**0/4**	1/1 (FP)	0/10^b^	2/4 (gilts) 0/3 (sows)	**0/3**		
Month 12	**0/3**	**0/3** **0/4**		**0/4**			2/5 (gilts) 0/48 (sows)^b^	**0/2**	1/1	1/1
Other *Brachyspira* spp.										
Month 6	**2/3 (*murdochii*)**	**2/3**	0/3 0/3	**0/4**	0/9 0/12	0/4	0/3	**0/3**		
Month 9		**0/3** **1/4 (*innocens*)**		**0/4**	0/3	1/10^b^ (*murdochii*)	0/5 (gilts) 0/5 (sows)	**0/3**		
Month 12	**2/3 (*pilosicoli*)**	**0/3** **0/4**		**0/4**			0/3	**0/2**	0/1	0/1
Clinical signs (diarrhoea) after eradication	**no**	**no**	yes	**no**	yes	yes	yes (mild, only in gilts)	**no**	yes	yes

^a^ Gilt rearing: from 10 weeks until pregnancy testing; sow farm: pigs raised until 10 weeks of age

^b^ Extra samples taken from clinically healthy pigs on known infected farms

^c^ FP fattening pigs

The eradication programme was successful in four farms. On these, no faecal samples tested positive for *B. hyodysenteriae* during the monitoring, and no diarrhoea was observed for at least 2 years after the programme. Two farms (farm 2; single-site farrow-to-finish herd and farm 4; finishing herd) had applied total depopulation and respected a vacancy period of at least 3 weeks. A third farm (farm 1; gilt rearing farm) practised partial depopulation, the rooms remained empty for 28 days, and they subsequently changed the source of the breeding gilts (also purchased at 14 weeks instead of 10 weeks). The fourth farm (farm 8; sow farm) practised partial depopulation, the stables remained empty for 3 weeks, and they used antimicrobial medication for 6 weeks.

The eradication programme was not successful in six farms. Clinical diarrhoea was present on five farms during the first 6 months after the eradication programme. On farm 7, diarrhoea occurred later: it was very mild and was present only in the gilts.

Three farms (3, 5 and 6) tested positive at the first sampling after 6 months; farm 7 tested positive at the second sampling after 9 months; and farms 9 and 10 were positive at the last sampling after 12 months (no earlier testing was done on these farms).

Two of the six farms (3 and 5) only used antimicrobial medication without partial depopulation. In farm 3, the entire farm was medicated, whereas in farm 5, the older fattening pigs were not medicated. In addition, in farm 5, the rooms of the fattening pigs had remained empty for only 3 days and other internal biosecurity measures such as cleaning and disinfection, separate equipment and boots for each age group were not properly practiced.

Four of the six farms where eradication was not successful (6, 7, 9 and 10) practiced partial depopulation. Farms 6, 9 and 10 did not use antimicrobial medication. The rooms had remained empty for 14 days (farms 6 and 9) or 21 days (farm 10). The cleaning and disinfection procedures were implemented properly, but in farm 10, rodent control was not successful and no separate equipment and boots for each age group was used. Farm 7 used antimicrobial medication, the cleaning and disinfection procedures and other internal biosecurity measures were considered to be appropriate, but the rooms had remained empty for only 10 days.

The results of the MLST typing of *B. hyodysenteriae* isolates and the antimicrobial susceptibility testing are shown in Table 4.

**Table 4:** Results of susceptibility testing and multilocus sequence typing (MLST) (PubMLST) of *Brachyspira hyodysenteriae* isolates obtained before and after the eradication program on the 10 farms. Farms with successful eradication are marked in bold

	Farm number									
	1	2	3	4	5	6	7	8	9	10
Before eradication										
Detected by …	**PCR**	**PCR, culture**	PCR, culture	**culture**	PCR	culture	PCR, culture	**culture**	culture	culture
Number of isolates	**0**	**1**	1	**1**	0	1	1	**1**	1	1
MIC-values (μg/ml) ^b^										
Tiamulin		**> 16**	0.16	**0.12**		2	2	**0.50**	> 16	> 16
Valnemulin		**> 16**	8	**0.12**		4	4	**1**	> 16	> 16
Tylvalosin		**> 128**	0.25	**16**		8	8	**128**	> 128	> 128
MLST type^a^			ST265				ST228		ST87	
Eradication successful	**yes**	**yes**	no	**yes**	no	no	no	**yes**	no	no
After eradication										
Detected by …			culture		PCR, culture	PCR	culture		culture	PCR
Number of isolates			1		1	0	1		1	0
MIC-value (μg/ml) ^b^										
Tiamulin			2		< 0.03		16		> 16	
Valnemulin			8		< 0.03		4		> 16	
Tylvalosin			0.12		1		32		128	
MLST type ^a^			ST265				ST264		ST87	

^a^ MLST analysis was only performed on farms where *B. hyodysenteriae* isolates could be obtained before and after the eradication program, allowing comparison of MLST types

^b^ Isolates were considered resistant if MIC-values (μg/ml) were higher than 2, 2 and 32 for tiamulin, valnemulin and tylvalosin, respectively [24, 25]

From three of the non-successful farms (3, 7 and 9), single *B. hyodysenteriae* isolates could be obtained before and after the eradication program. In two out of three farms, the same MLST type was found. In farm 7, the isolate obtained after the eradication programme showed a different MLST type (only one locus was different) than the isolate found before the eradication programme. In the other three non-successful farms (5, 6 and 10), only one *B. hyodysenteriae* isolate could be obtained and infection either before or after the eradication program was determined using PCR.

Three of the four farms that had used tiamulin during the eradication program were not successful (farms 3, 5 and 7). In farms 3 and 7, the MIC-values of the isolates obtained after eradication were significantly higher than those of the isolate obtained before isolation (farm 3: increase from 0.16 to 2; farm 7: increase from 2 to 16) (Table 4). This was not the case in farm 5 (MIC-value < 0.03). In farm 7, also tylvalosin was used during the program. The MIC-value for tylvalosin of the isolate obtained after eradication was also higher than the value obtained before the program (32 versus 8). The *B. hyodysenteriae* isolates obtained before and after the eradication program in farm 9 showed resistance to tiamulin, valnemulin and tylvalosin.

## Discussion

Different tailor-made eradication programmes were implemented in 10 farms with different characteristics that were infected with *B. hyodysenteriae.* Based upon monitoring during 1 year after the programme, eradication of *B. hyodysenteriae* was successful in 4 out of the 10 infected farms. Two years after eradication, none of the four farms had experienced any clinical problems related to *B. hyodysenteriae*.

The eradication programmes that were used can be classified into three major groups, namely total depopulation of animals on the farm, partial depopulation, and antimicrobial medication without depopulation. Most of the programmes were initiated in spring or summer, as environmental survival of *B. hyodysenteriae* is diminished during the warm season [30]. Every strategy was accompanied by different measures decreasing the risk for introduction and transmission of *B. hyodysenteriae* in the farms. The contribution of the voluntary implemented measures like the use of autogenous vaccines against *B. hyodysenteriae* and the addition of adsorbents or organic acids to the feed is not known, as the efficacy is uncertain and/or variable [14, 37]. None of the successful farms used autogenous vaccines, and nutritional changes were implemented both in successful and non-successful farms These measures might help to decrease the infection levels and shedding of *B. hyodysenteriae*, but they were insufficient as single measure to eliminate *B. hyodysenteriae* from infected farms.

Total depopulation was implemented in two farms (2, 4), and eradication was successful in both. Based on this result and the experience gained during the study, total depopulation followed by strict biosecurity measures is the easiest way and offers the greatest probability of successful eradication. This might also be the case for other pathogens. However, many farms were not willing or able to perform total depopulation because of financial reasons. Total depopulation was rather easy to implement in fattening farm 4, as the farm practiced all-in/all-out at barn level (only one barn) as a standard procedure. For the programme, the vacancy period between two successive batches of pigs was extended from one to 8 weeks.

Partial depopulation was practiced in six farms. Partial depopulation facilitates the implementation of biosecurity measures as it creates more space to move animals, to clean and disinfect, and to include a stand-empty period. Partial depopulation also reduces the number of animals to be treated. Two farms, a gilt rearing farm (farm 1) and a sow farm (farm 8) were successful using this strategy. Both farms strictly adhered to the different recommendations for preventing introduction of the pathogen in the herd and transmission within the herd. Farm 1 purchased gilts at 10 weeks of age, but changed the origin farm after the programme as it was suspected that the purchased animals from the original farm had introduced the infection onto the farm. Farm 8 did not purchase animals and had additionally applied antimicrobial medication for 42 days to all animals on the farm. This illustrates that farms that are not able to use total depopulation can also eliminate the pathogen from their farm if they strictly follow the biosecurity measures related to the partial depopulation programme and/or if an isolate is present that is susceptible to either one of the pleuromutilins or tylvalosin. This corroborates previous studies [18–20]. The two farms (1 and 8) were rather small (no fattening pigs), had a simple design (limited number of barns), and practiced all-in/all-out production. It can be expected that the likelihood of successful eradication using partial depopulation decreases when farms become larger and farm structure more complex e.g. single-site farrow-to-finish herds with complex structure of different barns and movement of age groups.

Four of the six farms that practiced partial depopulation, including three fattening pig farms and one sow farm, were however not successful. The precise reasons for the failed elimination are not clear, especially for farms 6 and 7 where most of the recommendations were implemented properly. However, the vacancy period for both farms might have been too short (10–14 days), and no antimicrobial medication was used in farm 6.

Although farms 9 and 10 indicated they were motivated to participate, they might not have followed the programme strictly enough. Antimicrobial treatment was not used as strains with acquired resistance to tiamulin, valnemulin and tylvalosin were present. There were no separate boots and equipment for the different age groups or barns, the vacancy period was rather short (14–21 days), and in farm 10, the rodent control was insufficient. Both farms continued to purchase feeder pigs from the same origin farms as before, although it is not sure whether this played a role as there was no evidence of *B. hyodysenteriae* infection on the origin farms.

Two farms decided to eliminate *B. hyodysenteriae* based on antimicrobial medication without depopulation. This strategy was possible as there was no evidence of *B. hyodysenteriae* strains that were resistant to tiamulin, valnemulin and tylvalosin prior to the programme. The fact that no depopulation was practiced was a major weakness as it hampered the implementation of proper cleaning, disinfection and stand-empty periods. The two farms practiced all-in/all-out at room level, but the stand-empty period between successive batches was short (3 days), leaving insufficient time for thorough drying of the environment [30]. Also the rodent control was insufficient on both farms. Further weaknesses in the programme of farm 5 were that the older fattening pigs (4–6 weeks prior to slaughter) were not medicated in order to limit expenses, no separate equipment and boots were used for the different age groups, and as the only farm out of the 10, the manure pits were not emptied and cleaned.

In two out of three farms where eradication was not successful, a *B. hyodysenteriae* strain was isolated before and after eradication with the same MLST pattern. This might suggest that the infection was not due to introduction of a new *B. hyodysenteriae* strain from outside the farm, but rather to persistence of the same strain in the herd. However, it cannot be excluded that the eradication programme as such was successful and that re-introduction of the strain occurred from an external source (e.g. via the origin farm where pigs were purchased, or via rodents from neighboring farms). Alternatively, finding a strain with a different MLST pattern before and after the eradication programme, as was the case in farm 7, might point to a new infection after the eradication programme, but it is also possible that different MLST type was already present on the farm or on the origin farm where the pigs were purchased [38]. Many more isolates from the farms should be typed before and after eradication in order to distinguish between an unsuccessful eradication programme and a successful eradication followed by reinfection. The study emphasizes the importance of *B. hyodysenteriae*-free stock for recruitment, and the importance of a control program for maintenance of a *B. hyodysenteriae*-free status of a herd.

The results of the susceptibility testing showed that in two of the three non-successful farms that used tiamulin, the MIC-values of the isolates obtained after eradication were significantly higher than those of the isolate obtained before isolation. Tiamulin medication that was used during the eradication programme may have favored development of higher-level resistance on these farms, since Card et al. [8] reported a new pleuromutilin resistance gene (tva(A) tiamulin valnemulin antibiotic resistance) that could lead to the selection of mutants with elevated MIC-values. The detection of strains with acquired resistance to tiamulin, valnemulin and tylvalosin in farm 9 that did not use antimicrobial medication during eradication, indicates that resistant strains may persist for a long time in farms. Antimicrobial susceptibility testing against lincomycin, tylosin and tetracyclines was not performed to limit expenses. The focus was on tiamulin, valnemulin and tylvalosin as these antimicrobials are mostly used against *Brachyspira* infections in Belgium.

The sampling protocol allowed a 90% level of confidence of assessing a farm population freedom of *B. hyodysenteriae* for farms 2 and 4. Due to a lower sample size, the observed level of confidence of freedom was 85% for farm 1 and 88% for farm 8. All four farms have been sampled at several occasions after the programme, and in contrast with the 6 unsuccessful farms, there was no evidence of clinical signs over two and half years after completion of the eradication programme. Given the low and intermittent shedding in farms with low infection levels [28], assessing the infection status based on faecal samples remains challenging. Other diagnostic tests such as the detection of serum antibodies against *B. hyodysenteriae* could also be used. However, serological tests generally have low specificity and/or sensitivity. An ELISA using the H114 surface protein correctly detected apparently healthy farms that subsequently were confirmed to contain pigs colonized with *B. hyodysenteriae* [39]. However, also 14 out of 18 unsuspected farms where no *B. hyodysenteriae* was confirmed showed positive serology. Further research is needed to develop accurate methods that can be used for routine screening of pig farms to detect animals with low levels of colonization.

Fifty infected farms had been contacted by their herd veterinarians and asked for possible participation in the study. However, most of them did not want to collaborate for the different reasons mentioned above, and in the end, only 10 farms were willing to participate. This is somewhat surprising as eradication leads to better performance, less antimicrobial use and is financially beneficial in rather short time [20]. Also a recent interview-based study by Cadetg et al. [40] showed that a majority of Swiss farmers that had practiced eradication were satisfied with the outcome.

## Conclusions

Eradication of *B. hyodysenteriae* was successful in 4 of 10 infected farms, with two using total depopulation and two using partial depopulation. Eradication was not successful on the other six farms, including four using a partial depopulation programme, and two using antimicrobial medication without medication. The four successful farms succeeded in strictly implementing all the proposed biosecurity measures such as thorough cleaning and disinfection, a vacancy period of at least 21 days, and efficient rodent control, whereas this was not always the case in the farms where the eradication was not successful. Apart from the type of eradication programme and the structure of the farm, the motivation of the farmer to strictly implement all the different measures of the programme and to maintain high biosecurity standards afterwards are essential to eradicate this economically important pathogen from the herds.

## Data Availability

The datasets supporting the conclusions of this article are available upon request of the corresponding author.
